# COVID-19, vaccination and migraine: Causal association or epiphenomenon?

**DOI:** 10.1371/journal.pone.0308151

**Published:** 2024-08-19

**Authors:** Hailun Jiang, Chao Zhang, Xianggang Meng, Shihao Chi, Danqi Huang, Shizhe Deng, Guang Tian, Zhihong Meng

**Affiliations:** 1 Department of acupuncture, First Teaching Hospital of Tianjin University of Traditional Chinese Medicine, Tianjin, China; 2 Graduate School, Tianjin University of Traditional Chinese Medicine, Tianjin, China; 3 Department of acupuncture, National Clinical Research Center for Chinese Medicine Acupuncture and Moxibustion, Tianjin, China; 4 Office for Cancer Screening, National Cancer Center/National Clinical Research Center for Cancer/Cancer Hospital, Chinese Academy of Medical Sciences and Peking Union Medical College, Beijing, China; University of Turin, ITALY

## Abstract

**Background:**

Diverse studies have revealed discrepant evidence concerning the causal association between Corona Virus Disease 2019 (COVID-19) and COVID-19 vaccination in relation to migraines. Investigating the correlation between the former two factors and migraines can facilitate policymakers in the precise formulation of comprehensive post-pandemic interventions while urging the populace to adopt a judicious perspective on COVID-19 vaccination.

**Methods:**

We undertook a Mendelian randomization (MR) study. The primary assessment of the causal relationship between the three different COVID-19 exposures and migraine was conducted using the standard inverse variance weighted (IVW) approach. In the supplementary analysis, we also employed two methodologies: the weighted median estimator (WME) and the MR-Egger regression. Ultimately, the reliability and stability of the outcomes were assessed via Cochran’s Q test, the leave-one-out method, the MR-Egger intercept test, and the MR pleiotropy residual sum and outlier (MR-PRESSO) test.

**Results:**

The results indicate an absence of correlation between genetically predicted COVID-19 (①Very severe respiratory confirmed COVID-19: odds ratio [OR], 1.0000881; 95%CI, 0.999748–1.000428; p = 0.6118; ②Hospitalized COVID-19: OR, 1.000024; 95%CI, 0.9994893–1.000559; p = 0.931;③SARS-CoV-2 infection: OR, 1.000358; 95%CI, 0.999023–1.001695; p = 0.5993) and the risk of migraine. Furthermore, the MR-Egger regression and WME also yielded no evidence of COVID-19 elevating the risk of migraine occurrence. Sensitivity analysis affirmed the robustness and consistency of all outcomes.

**Conclusions:**

The results of this study do not offer genetic evidence to substantiate a causal relationship between COVID-19 and migraines. Thus, the deduction drawn from COVID-19 genetic data is that COVID-19 vaccination is unlikely to exert an impact on the occurrence of migraines, though this conclusion warrants further investigation.

## Introduction

The worldwide dissemination and propagation of Severe Acute Respiratory Syndrome Coronavirus 2 (SARS-CoV-2) have engendered substantial repercussions on the physical and psychological well-being of individuals. As per the weekly updated epidemiological reports from the World Health Organization (WHO), up to September 24, 2023, the cumulative documented cases worldwide have exceeded 770 million, with fatalities surpassing 6 million [1]. It can be comprehended that during the outbreak phase of the pandemic, the majority of research has been concentrated on the potential pathophysiology of COVID-19 and the management of acute cases during hospitalization. Subsequently, with the proliferation of vaccines and the diminished virulence of the Omicron variant, what has become more prevalent among the population is asymptomatic infection. Consequently, the global scenario is gradually transitioning from an emergent phase to a post-pandemic era [2]. In the post-pandemic era, there has been a decline in testing for and reporting SARS-CoV-2 infections. Consequently, the research focus has shifted from infection rates to a second pandemic known as "long haul COVID" [3]. Presently, multiple pieces of evidence from evidence-based medicine suggest that individuals who have been infected with SARS-CoV-2 are likely to experience one or several lingering sequelae and/or medical complications following their initial recovery. These enduring, protracted symptoms, which can persist for several months or even years, inflict prolonged distress upon affected individuals. The most prevalent psychological and neurological manifestations encompass fatigue, headache, cognitive impairment, anxiety, and depression [4–6]. The long-term neurological sequelae of COVID-19 refer to a series of symptoms such as sustained inattention, headaches, sensory disturbances, and depression that persist for an extended duration following the acute phase of COVID-19 [7].

Migraine, a common disease in the department of neurology, is categorized as a form of chronic headache, accounting for 88.2% (ranging from 60.7% to 97.7%) of the overall burden of headache disorders [8]. Furthermore, it stands as the second-largest contributor to global neurological disability-adjusted life years (DALYs) worldwide, and it is even more severe among younger and middle-aged individuals [9]. In clinical practice, neurologists have observed the existence of various types of "post-COVID headaches". Particularly notable is the persistent chronic headache characterized by migraine-like attacks. This type of headache can manifest as either a sudden worsening of preexisting migraines, or it may occur in patients with no prior history of migraines [10]. Moreover, vaccines capable of intracellular ribosomal utilization for translating into the spike S protein have prompted public apprehension concerning migraines following COVID-19 vaccination [11–13]. Previous clinical randomized controlled trials (RCTs) have also indicated that headaches constitute one of the most prevalent adverse reactions following vaccination [14, 15]. Nonetheless, recent clinical studies propose that the exacerbation of migraines due to SARS-CoV-2 infection and COVID-19 vaccination might be predominantly attributed to a nocebo effect within the general populace [16]. The diverse outcomes indicate a need for forthcoming investigations to delve deeper into the relationship between COVID-19 (vaccination) and migraines.

In addition to the aforementioned clinical evidence, several laboratory studies have also provided evidence of the SARS-CoV-2’s potential ability to develop long-term neurological sequelae [17]. Present laboratory investigations reveal that high levels of SARS-CoV-2 Ribonucleic Acid (RNA) are observable within the trigeminal ganglia of COVID-19 patients through Real-Time Quantitative Polymerase Chain Reaction (RT-qPCR) testing [18]. Consequently, the researchers proposed that the occurrence and deterioration of migraine may, on one hand, be associated with the abnormal activation of the trigeminal neurovascular system by SARS-CoV-2 or vaccines expressing SARS-CoV-2 spike protein, mediated through an immune-inflammatory response, leading to vascular and neural damage [19–22]. On the other hand, it may be related to SARS-CoV-2’s neurotropic properties, allowing it to bind to hACE2 receptors on cell surfaces via the spike S protein in the virus, thereby facilitating neuronal-neuronal, neuronal-glia, and glia-glia fusion, and these fused neurons exhibit severe impairment of neuronal activity and may even permanently alter neuronal circuits and function [17]. However, translating the laboratory research findings into clinical implications still faces certain limitations. Currently, it remains inconclusive that the immune-inflammatory response and compromised neuronal activity induced by the SARS-CoV-2 or the vaccines that code for the SARS-CoV-2 spike protein constitute the foundational etiology of post-COVID-19 migraine. Moreover, the current level of clinical evidence available is not high, making it remains challenging to establish a causal relationship between COVID-19 (and COVID-19 vaccination) and traits related to migraines.

In the absence of large-scale and high-quality RCTs, Mendelian randomization (MR) has emerged as a pivotal alternative strategy for predicting disease risk factors. This methodology harnesses irrevocable genetic variations to minimize confounding biases to the greatest extent possible, thereby ascertaining whether the association of coexisting traits (COVID-19 and migraine) is consistent with a causal effect [23]. Although there have been partial RCTs focusing on COVID-19 vaccination, the challenge of avoiding bias persists, and long-term follow-up is difficult to achieve. Consequently, establishing a causal connection between persistent migraines and vaccination remains a formidable undertaking. From the perspective of the evidence hierarchy pyramid, MR studies occupy a loftier echelon of evidence compared to observational research [24]. Observational studies can effectively mitigate bias caused by measured confounding factors through techniques such as multiple regression, stratified analysis, and propensity scores. Furthermore, MR analysis extends its utility by addressing unmeasured (or unknown) confounding factors that traditional statistical methods cannot deal with. In light of the lack of large-scale Genome-Wide Association Study (GWAS) data pertaining to COVID-19 vaccination, we leverage COVID-19 GWAS data as a bridge to investigate the potential causal impact of three COVID-19 features (susceptibility, hospitalization, and severity) on migraine, and deduce the correlation between COVID-19 vaccination and the incidence of migraines.

## Materials and methods

**Fig 1:**
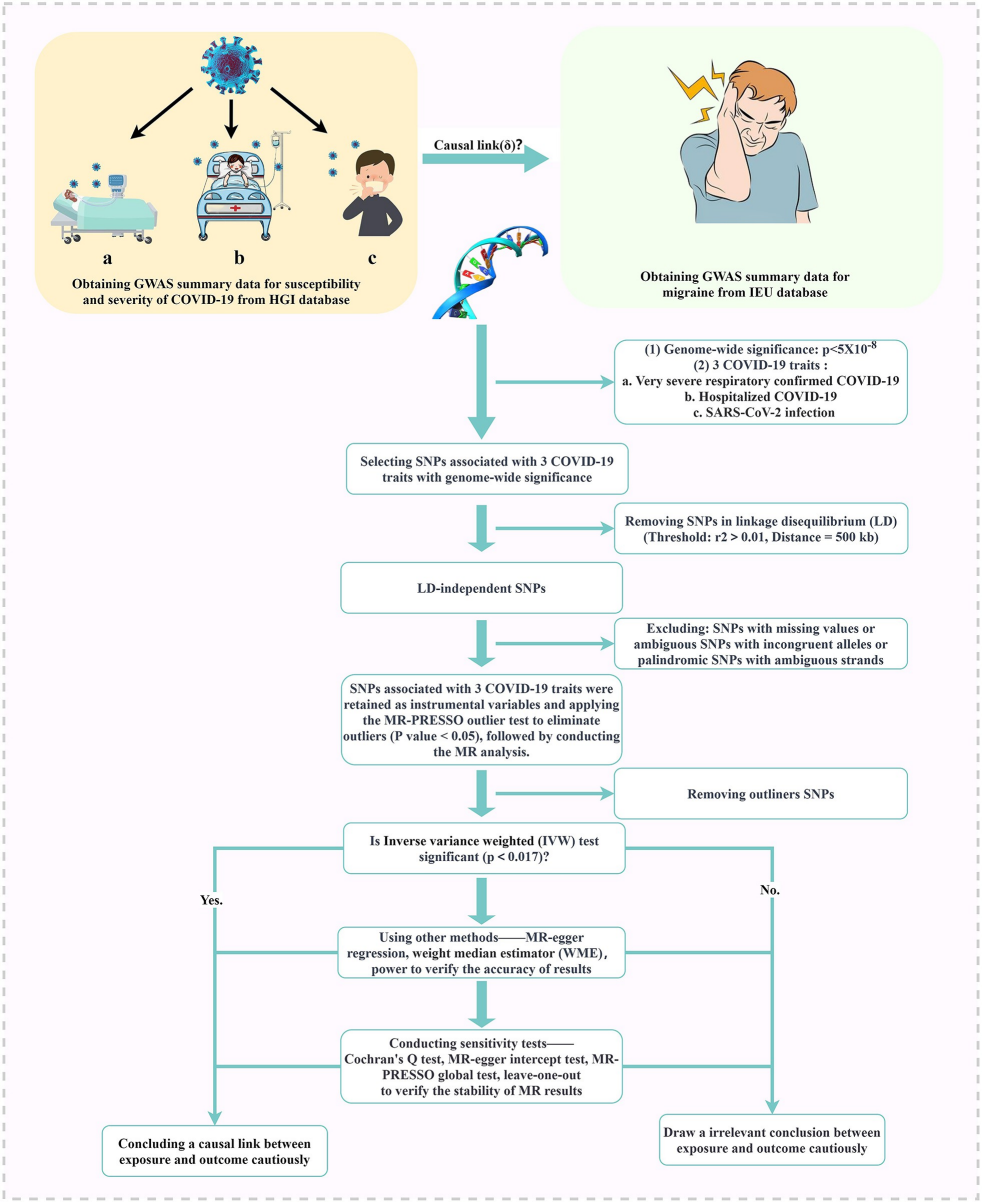
Flowchart depicting the procedural course of this study.

Both datasets utilized in this MR analysis were derived from publicly available aggregated GWAS data, obviating the need for additional ethical approval or informed consent. The STROBE-MR checklist has been included as the supplementary file (S1 Table) [25], and an illustrative flow chart delineating the study’s procedural course is available in Fig 1.

### Exposure and outcome

In this MR analysis, three COVID-19 traits were employed as exposure factors: a. Very severe respiratory confirmed COVID-19: critically severe cases of COVID-19, characterized by individuals necessitating hospital-based respiratory support or experiencing mortality due to the disease; b. Hospitalized COVID-19: cases of moderate or severe COVID-19 defined as those participants who were hospitalized due to symptoms associated with the infection; c. SARS-CoV-2 infection: all reported cases of SARS-CoV-2 infection, regardless of symptomatic manifestation [26, 27]. The outcome factor comprises individuals who meet the international classification of diseases(ICD)-10 diagnostic criteria for migraine. All participants in the control group are drawn from the general population and do not manifest the corresponding traits.

### The core assumptions of Mendelian randomization

**Fig 2:**
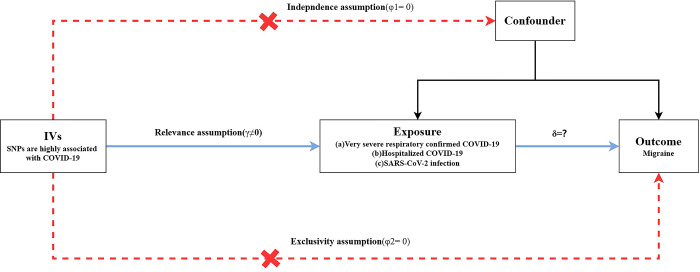
The core assumptions of Mendelian randomization.

In this study, we conducted a two-sample MR analysis to explore the causal relationship (δ =?) between exposure factors ((1) Very severe respiratory confirmed COVID-19; (2) Hospitalized COVID-19; (3) SARS-CoV-2infection and the outcome factor (migraine). This study adheres to the three core assumptions of MR [28], as depicted in Fig 2: (1) Exclusivity Assumption: Genetic variations (single-nucleotide polymorphisms (SNPs)) used as IVs are strongly associated with the exposure factors (γ≠0); (2) The independence assumption: SNPs are independent of any confounding factors affecting the exposure-outcome association (φ1 = 0); (3) Relevance Assumption: SNPs do not directly influence the occurrence of the outcome and can only affect the outcome through the exposure factors (φ2 = 0).

### Data source

The GWAS data for exposure factors were obtained through The COVID-19 Host Genetics Initiative (https://www.covid19hg.org/), which includes: (1) Very severe respiratory confirmed COVID-19: (13,769 cases and 1,072,442 controls); (2) Hospitalized COVID-19: (32,519 cases and 2,062,805 controls); (3) SARS-CoV-2 infection: (122,616 cases and 2,475,240 controls). The COVID-19 datasets were acquired from the COVID-19 Host Genetics Initiative GWAS Round Seven, released on April 8, 2022. The GWAS data for the outcome factor (migraine) were obtained through the IEU OpenGWAS project (https://gwas.mrcieu.ac.uk/), consisting of 1,072 cases and 360,122 controls. Therefore, GWAS data from distinct databases demonstrate either negligible or exceedingly limited sample overlap, while ensuring that all participants originate from European populations. Please refer to Table 1 for a summary of the GWAS results.

**Table 1:** Comprehensive summary of the GWAS results.

Phenotype	Sample size	SNPs(n)	Cases (n)	Controls (n)	Population
Very severe respiratory confirmed COVID-19	1,086,211	12,195,848	13,769	1,072,442	European
Hospitalized COVID-19	2,095,324	12,484,092	32,519	2,062,805	European
SARS-CoV-2 infection	2,597,856	14,498,647	122,616	2,475,240	European
Migraine	361,194	9,633,338	1,072	360,122	European

### Selection of the genetic instruments

Firstly, it is imperative to ensure a robust and strong correlation between the primary genetic instruments and the exposure factors. The selected SNPs must individually surpass the genome-wide significance threshold (p < 5×10^−8^) in three distinct traits: a. Very severe respiratory confirmed COVID-19; b. Hospitalized COVID-19; c. SARS-CoV-2 infection. Subsequently, to select SNPs that are sufficiently distant to approximate independence (setting: kb = 500, r ^2^ = 0.01), SNPs in a state of linkage disequilibrium (LD) (having a correlation > 0.01 with the most significant SNP within a distance of 500 kb) shall be systematically pruned [29]. Thirdly, to ensure the alignment of the selected SNPs in exposure and outcome GWAS, ambiguous SNPs with incongruent allelic variants or palindromic SNPs with ambiguous strands will be subjected to filtration and subsequently discarded. Furthermore, SNPs with missing data in the dataset will also be removed. Lastly, to mitigate weak instrument bias, the instrumental strength of each SNP selected through the preceding three steps will be estimated using the F-statistic. SNPs with an F-statistic > 10 will ultimately be designated as primary genetic instruments [30]. R^2^ and F-statistics were computed as follows:

R^2^ = 2*(1-MAF) * MAF *β^2^, where MAF represents the minor allele frequency, and when calculating R^2^, MAF can be considered equivalent to the effect allele frequency (EAF). thus:

R^2^ = 2*(1-EAF)*EAF*β^2^

$x=(\frac{R^{2}}{1-R^{2}})(\frac{N-K-1}{K})$

β, the genetic estimate of exposure per IV; N, the sample size (comprising both cases and controls); k, the number of IVs; EAF, the effect allele frequency; SE, the standard error [31].

### Statistical analysis

This study will employ methodologies such as IVW, WME, the MR-Egger Regression, and MR-PRESSO to assess the causal relationship between exposure and outcomes in MR analysis. Primary Outcome: The random-effects IVW model is the most widely applied in MR studies. This model assumes that all SNPs are valid IVs, and the overall effect is estimated as the inverse variance-weighted average of the effects of the IVs. In the absence of horizontal pleiotropy bias, the IVW model exhibits greater statistical power, thereby furnishing dependable causal estimates [32, 33]. Secondary Outcome: WME and MR-Egger Regression methods, employed as supplementary methods to complement IVW estimates, provide valuable sensitivity analyses in detecting violations of the instrumental variable assumption. However, WME is more likely to achieve consistent causal assessments when over 50% of the weight is derived from valid instrumental variables [34], while the MR-Egger method typically yields causal estimates with standard errors greater than those of the IVW method [35]. In both respects, under the condition of no horizontal pleiotropy, the random-effects IVW model exhibits superior capability in obtaining unbiased estimates compared to the WME and the MR-Egger Regression methods [34, 36]. Based on the aforementioned rationale, we have elected to employ the random-effects IVW model as the primary computational approach for the primary outcome. The primary and secondary outcomes are expressed as ORs accompanied by their respective 95% CIs. To obtain a more rigorous interpretation of causality, statistical significance will be determined with Bonferroni correction, where a significance threshold of p < 0.05/3 (= 0.017) is deemed statistically significant, while p-values falling between 0.017 and 0.05 are considered to have nominal causal significance.

Subsequently, sensitivity tests are conducted to ensure the stability of the MR results. Firstly, Cochran’s Q test was employed to assess the heterogeneity of individual causal effects (If p > 0.05, indicating the absence of heterogeneity, the causal estimates are considered more reliable). Secondly, the run_mr_presso function was employed to conduct the MR-PRESSO outlier test. This involved calculating the sum of squared residuals, which represents the distance of each SNP to the fitted line when that SNP is excluded. This was done to detect potential outlier SNPs. If p > 0.05, it was considered that there are no outlier SNPs. If p < 0.05, the identified outlier SNPs needed to be removed to correct for horizontal pleiotropy, and the final reporting includes the corrected causal effects [37]. Thirdly, the MR-Egger intercept and the MR-PRESSO global test were employed to assess the overall level of horizontal pleiotropy. A threshold of p > 0.05 was considered as an absence of horizontal pleiotropy. Lastly, a leave-one-out analysis was conducted to examine result stability. This involved systematically removing individual SNPs one by one and calculating the remaining SNP effects. This analysis was performed to assess whether any chance effects driven by individual SNPs exist and to clarify the influence of individual differences on the observed associations.

All analyses in this study were conducted using R statistical software (version 4.3.1) and the R packages "TwoSampleMR" and "MRPRESSO."

## Results

### Results of SNPs and the weak IV test

This study analyzed the causal relationship between COVID-19 and migraine through the examination of three distinct exposure factors. Following the removal of SNPs with missing values, we ultimately identified 40 independent SNPs associated with Very severe respiratory confirmed COVID-19, 43 linked to Hospitalized COVID-19, and 23 significantly correlated with SARS-CoV-2 infection as IVs. It is noteworthy that the F-statistics for all these SNPs exceeded 10. These findings indicate the absence of potential weak instrument bias, thereby fulfilling the MR’s relevance assumption.

See S2 Table for more information on the Results of SNPs and the weak IV test.

### Causal effects of COVID-19 on risk of migraine

**Fig 3:**
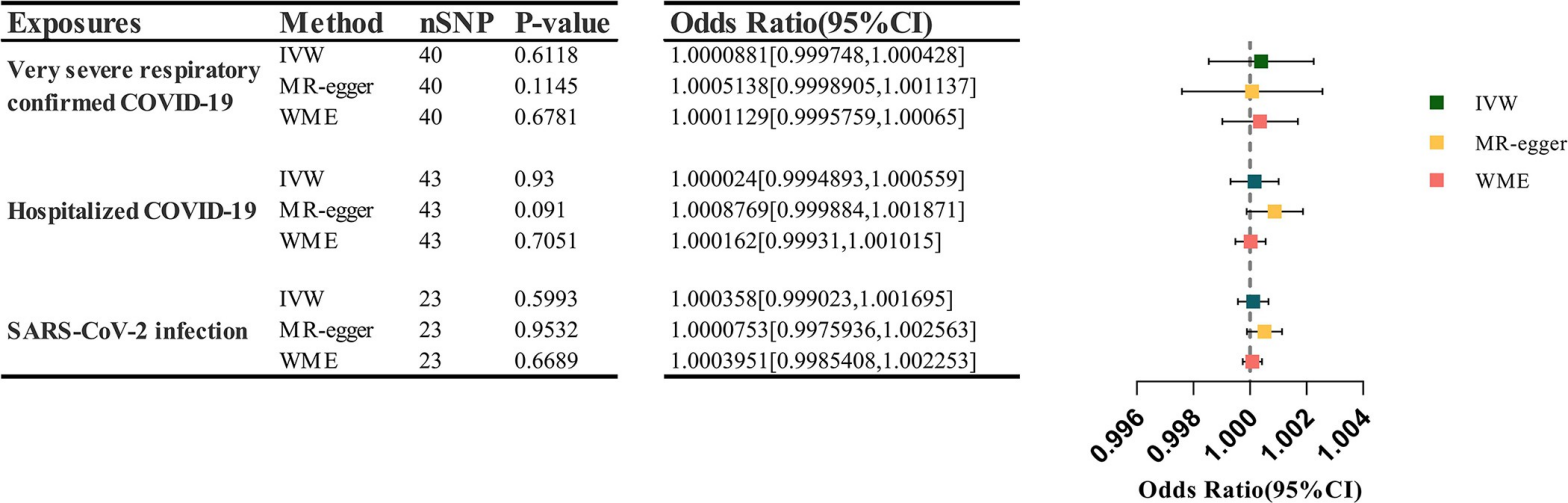
Causal effects of COVID-19 on risk of migraine.

The outcomes obtained from all three MR analysis methods we employed consistently indicate that the three exposures (Very Severe Respiratory Confirmed COVID-19, Hospitalized COVID-19, and SARS-CoV-2 infection) do not elevate the risk of migraine in patients. There exists no causal association between exposures and the outcome, and these results are summarized in Table 2 and Fig 3. Furthermore, based on the MR analysis results, we have generated a Forest plot, which allows for the observation of the effects driven by each SNP on the outcome. These results are discernible in S1 Fig.

**Table 2:** Mendelian randomization analyses of susceptibility and severity of COVID-19 on the risk of migraine.

Exposures	Method	SNP (n)	β	Se	P-value	Power
Very severe respiratory confirmed COVID-19	IVW	40	8.80887e-05	0.000173551	0.6118	0.05
	MR—egger	40	0.000513637	0.000317933	0.1145	/
	WME	40	0.000112914	0.000272013	0.6781	/
Hospitalized COVID-19	IVW	43	2.397375e-05	0.0002728769	0.9310	0.05
	MR—egger	43	8.765394e-04	0.0005064229	0.0910	/
	WME	43	1.619777e-04	0.0004279716	0.7051	/
SARS-CoV-2 infection	IVW	23	3.579226e-04	0.0006813030	0.5993	0.05
	MR—egger	23	7.530991e-05	0.0012676409	0.9532	/
	WME	23	3.949928e-04	0.0009235277	0.6689	/

IVW: Inverse variance weighted; WME: weight median estimator

### Evaluation of reliability

**Fig 4:**
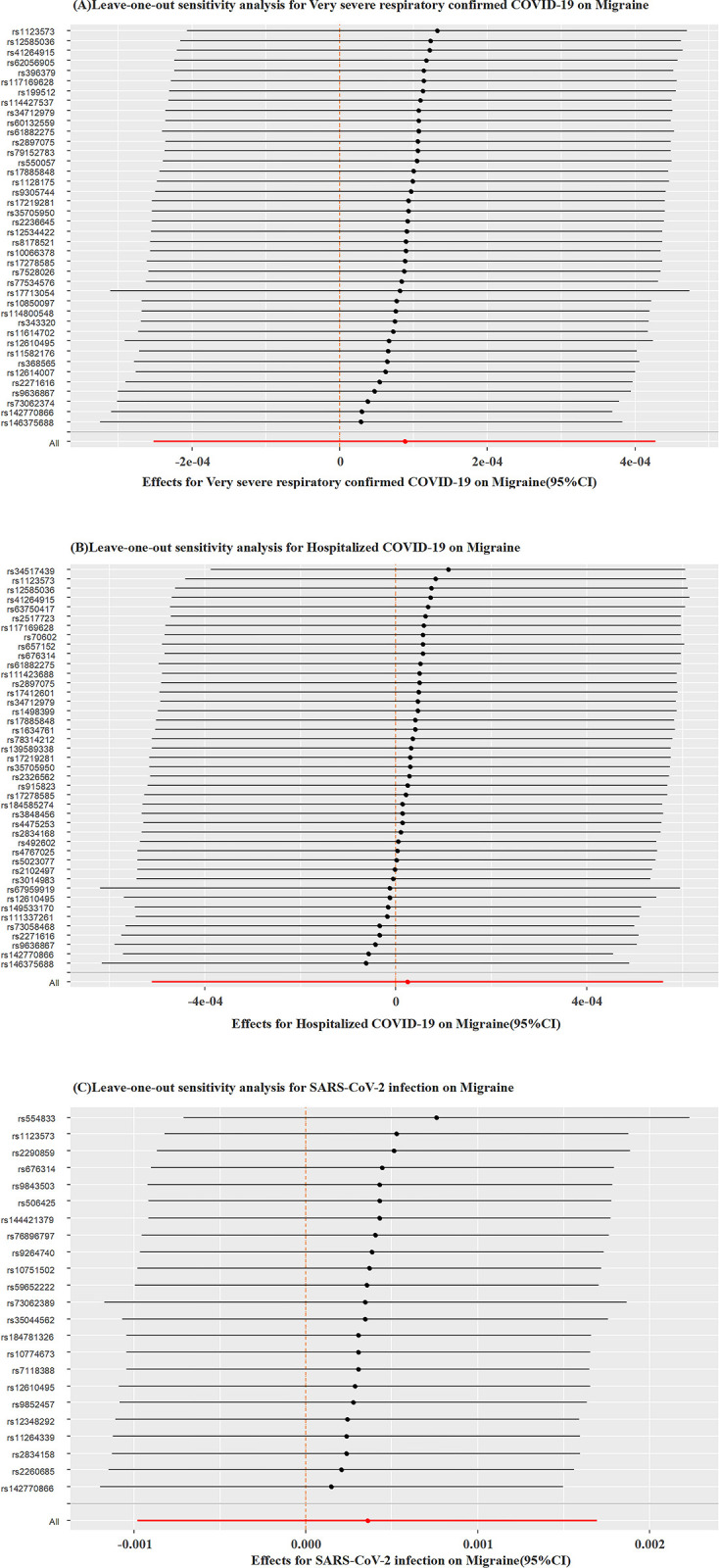
(A-C): Leave-one-out sensitivity analysis between the three different COVID-19 exposures and migraine. Note: Red lines represent estimates from IVW tests.

**Fig 5:**
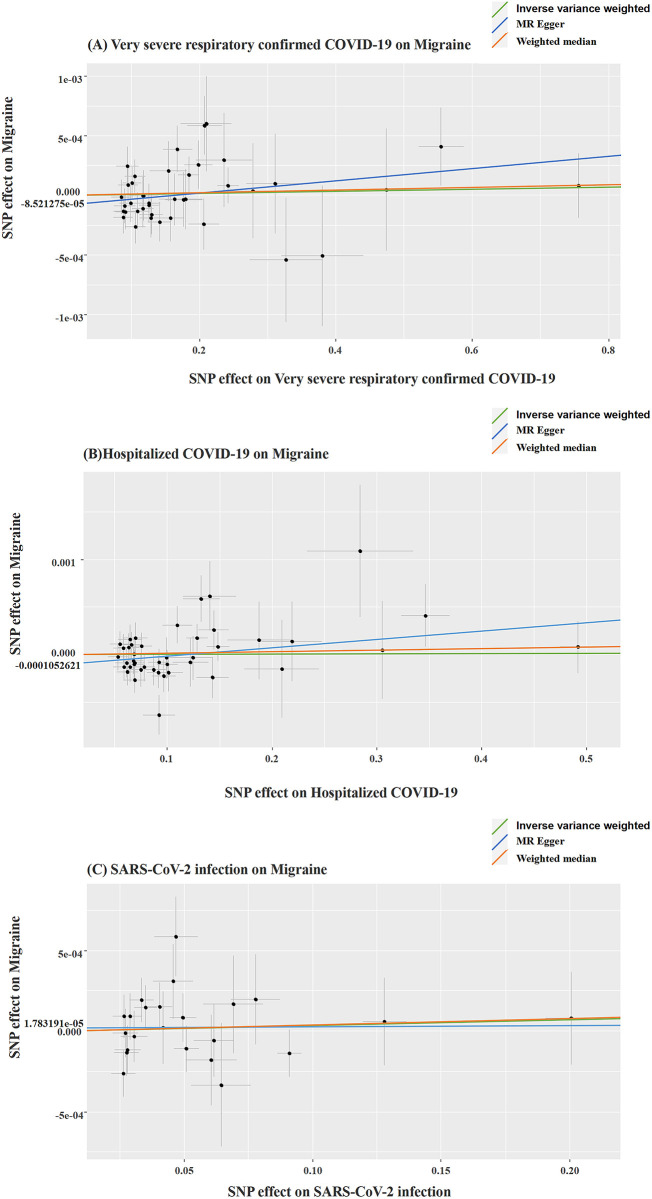
(A-C): Scatter Plots of Causality. Note: The scatter plots depict causality relationships, with each line’s slope corresponding to the estimated Mendelian Randomization (MR) effect in various models.

We assessed the heterogeneity of causal effects between the three exposures and outcomes using Cochran’s Q test, which did not reveal statistically significant heterogeneity at the level of statistical significance. Furthermore, the MR-PRESSO outlier test conducted using the run_mr_presso function did not detect any outliers, as summarized in Table 3. Additionally, we employed a leave-one-out analysis, gradually removing each SNP, and observed that the results with the remaining SNPs were largely consistent with the original results. This analysis helped eliminate the possibility of chance effects driven by any single SNP, as depicted in Fig 4A–4C. A visual examination of the funnel plot revealed basic symmetry, as displayed in S2 Fig. The above results collectively demonstrate a high level of stability in the MR analysis results, making them convincing and robust. Furthermore, the results of the MR-Egger intercept test and The MR-PRESSO global test (p > 0.05) provided evidence against the presence of overall horizontal pleiotropy, as depicted in Fig 5A–5C and Table 3.

**Table 3:** Reliability test of MR analysis results.

Exposure	Outcome	MR—egger		Cochran’s Q test		MR-PRESSO outlier test	MR-PRESSO global test
		Intercept	P-value	P-value (IVW)	P-value (MR—egger)	Outlier	P-value
Very severe respiratory confirmed COVID-19	Migraine	-8.521275e-05	0.1202	0.4285	0.4968	Null	0.4607
Hospitalized COVID-19		-0.0001052621	0.05530904	0.2051	0.3081	Null	0.2273
SARS-CoV-2 infection		1.783191e-05	0.7940	0.5151	0.4577	Null	0.539

## Discussion

Given the considerable prevalence of individuals worldwide who have been afflicted by SARS-CoV-2 and the potential for an enduring post-pandemic era, it becomes imperative to expeditiously develop pertinent prophylactic and intervention strategies. This entails strengthening the management of the people manifesting chronic conditions after COVID-19, encouraging the public to adopt a judicious approach to COVID-19 vaccination, thereby mitigating the burden on healthcare expenses and other societal exigencies. To our knowledge, this is the first study that utilizes large-scale GWAS data in European populations to explore the causal impact of COVID-19 severity and susceptibility on migraines through a two-sample MR approach.

This study assessed the impact of COVID-19 on migraines, and in our two-sample MR analysis, we did not find strong evidence to directly attribute the occurrence of post-COVID-19 migraines to COVID-19 infection. Therefore, we have further deduced that the manifestation of migraine attacks is not directly associated with vaccination. However, other intermediate factors may be involved in the development of migraines. During lockdowns and isolation, factors such as negative psychological and behavioral changes (such as abrupt lifestyle alterations, unemployment, depression, anxiety, insomnia, etc.), limited access to treatment medications, and the inability to participate in face-to-face counseling may have played an essential role in the development and progression of migraines [38–40]. Additionally, activation of the trigeminal vascular system may be a significant factor in the occurrence of migraines. SARS-CoV-2 infection and vaccination can lead to neuroinflammation and abnormal neural immune responses [41], characterized by widespread activation of CD4+T-cells, CD8+T-cells, microglial cells and abnormal proliferation of astrocytes [42]. These abnormally activated immunocytes can secrete various cytokines and inflammatory mediators to modulate central nervous system inflammation [43]. Factors such as interleukin (IL)-1β, nuclear factor kappa-B(NF-κB), prostaglandin E2 (PGE2), and nitric oxide (NO) play roles in migraine associated with activation and sensitization of the trigeminovascular system [44]. In several clinical studies related to migraines, it has been observed that migraine patients have higher levels of inflammatory factors in their plasma compared to individuals without migraines [45]. SARS-CoV-2 and COVID-19 vaccination can induce humoral and cellular immune responses, triggering a systemic state of high inflammation, often referred to as a "cytokine storm", resulting in elevated levels of inflammatory factors such as IL-1β, IL-6, tumor necrosis factor(TNF)-α [46, 47], and notably, an increase in IL-6 levels, which is considered a key element in the pathophysiology of migraines [48–50]. Therefore, the systemic high-inflammatory state induced by SARS-CoV-2 or vaccine may also be one of the intermediate factors promoting the occurrence of migraines in COVID-19. Symptoms such as fatigue, muscle pain, cough, and headache are typically short-term manifestations following COVID-19 [51]. Usually, as systemic inflammation recedes, migraines in individuals who have been infected with the SARS-CoV-2 or vaccinated tend to alleviate. This may lead to a decrease in the direct correlation between the former two and migraines, which could be a significant contributing factor to the inconsistencies observed between Mendelian randomization studies and clinical research. Moreover, extensive vascular dysfunction is also a significant factor contributing to neurological symptoms [7], and the angiotensin-converting enzyme 2 (ACE2) receptor is expressed in vascular endothelium [52]. When SARS-CoV-2 interacts with its ACE2 receptor, it can lead to endothelial inflammation, causing diffuse endothelial inflammation [52]. Endothelial dysfunction can lead to migraines as well [53]. There is another viewpoint that suggests SARS-CoV-2 exhibits neurotropic characteristics and can directly invade the peripheral endings of the trigeminal nerve in the nasal cavity, potentially leading to migraines [19]. Currently, we cannot rule out whether the factors mentioned above play an essential role in the development of post-COVID-19 migraines.

Discrepancies between MR and observational studies are common. Some previous clinical observational studies have shown negative impacts of COVID-19 infection and the administration of COVID-19 vaccines on the evolution of migraines, suggesting that it might worsen the condition in migraine patients [13, 21, 39, 54–56] and could be a potential trigger for migraine onset [57]. The significant differences between the results of our MR analysis and previous observational epidemiological studies are likely due to unmeasured confounding factors in the observational studies. This could also be attributed to the presence of a reverse placebo effect following infection with SARS-CoV-2 and COVID-19 vaccination, resulting in the amplification of complications associated with SARS-CoV-2 and adverse reactions to COVID-19 vaccination [16].

Indeed, the current study also has certain limitations. Firstly, limitations related to the original data: the data used in this study were derived from previously conducted research uploaded to public databases, and conducting further stratified analyses or adjusting for additional covariates beyond the currently available data may not be feasible. Secondly, limitations in generalizability: the dataset we selected only included European populations, limiting the generalizability of the results to non-European populations. The same genetic variants may have different effects in different populations, which may limit the applicability of the study’s findings to other groups. Thirdly, no further analysis of the selected SNPs with the second phenotype: Considering that the relationship between most SNPs and the second phenotype is still to be explored and validated, this study did not screen for SNPs that might be related to the second phenotype on the PhenoScanner website. Fourthly, a limited number of IVs: Building effective IVs can be challenging, and the power of MR is currently low, with only a small number of SNPs explaining variations in the COVID-19 traits selected, which may have influenced the results. It’s important to acknowledge that a higher number of valid IVs can increase the statistical power and reliability of MR analyses. Lastly, there is a lack of GWAS data for COVID-19 vaccination: COVID-19 vaccination triggers immune responses, primarily involving cross-reactivity associated with the spike protein. Currently, causality between COVID-19 vaccination and migraines can only be inferred from GWAS data related to COVID-19, and this inference carries certain limitations.

## Conclusion

We cautiously conclude that after Bonferroni correction, there is insufficient evidence to establish a causal relationship between COVID-19 and migraine-related traits. Even in nominal terms, all the MR results we obtained do not indicate a causal effect between COVID-19 and migraine-related traits. However, whether these conclusions can be generalized to individuals receiving COVID-19 vaccination still requires validation in larger-scale clinical research. Furthermore, while our current study does not provide evidence for a direct causal effect of COVID-19 on migraines, it remains essential to acknowledge that the association between migraines and COVID-19 (and COVID-19 vaccination) may operate through alternative shared pathways(e.g., lifestyle modifications, inflammation, etc.). Our study still emphasizes the importance of timely intervention and management for individuals with post-COVID migraines and highlights the need for society and families to address the physical and mental health concerns of post-COVID individuals. Simultaneously, it also advocates for fostering a proper attitude towards vaccination. This, in the end, leads to a reduction in the burden on society and families and alleviating the strain on healthcare resources.

## Supporting information

S1 Fig Forest plots of the effects driven by each SNP on the migraine. (TIF)

S2 Fig Funnel plot of SNPs. (TIF)

S1 Table STROBE-MR-checklist. (DOCX)

S2 Table Details of specific single nucleotide polymorphisms for each covid-19 trait. (DOCX)
