# Supplementary figures and images for: COVID-19, vaccination and migraine: Causal association or epiphenomenon?
Source: PLoS One. 2024 Aug 19;19(8):e0308151. doi: 10.1371/journal.pone.0308151 (PMC11333006; doi:10.1371/journal.pone.0308151)

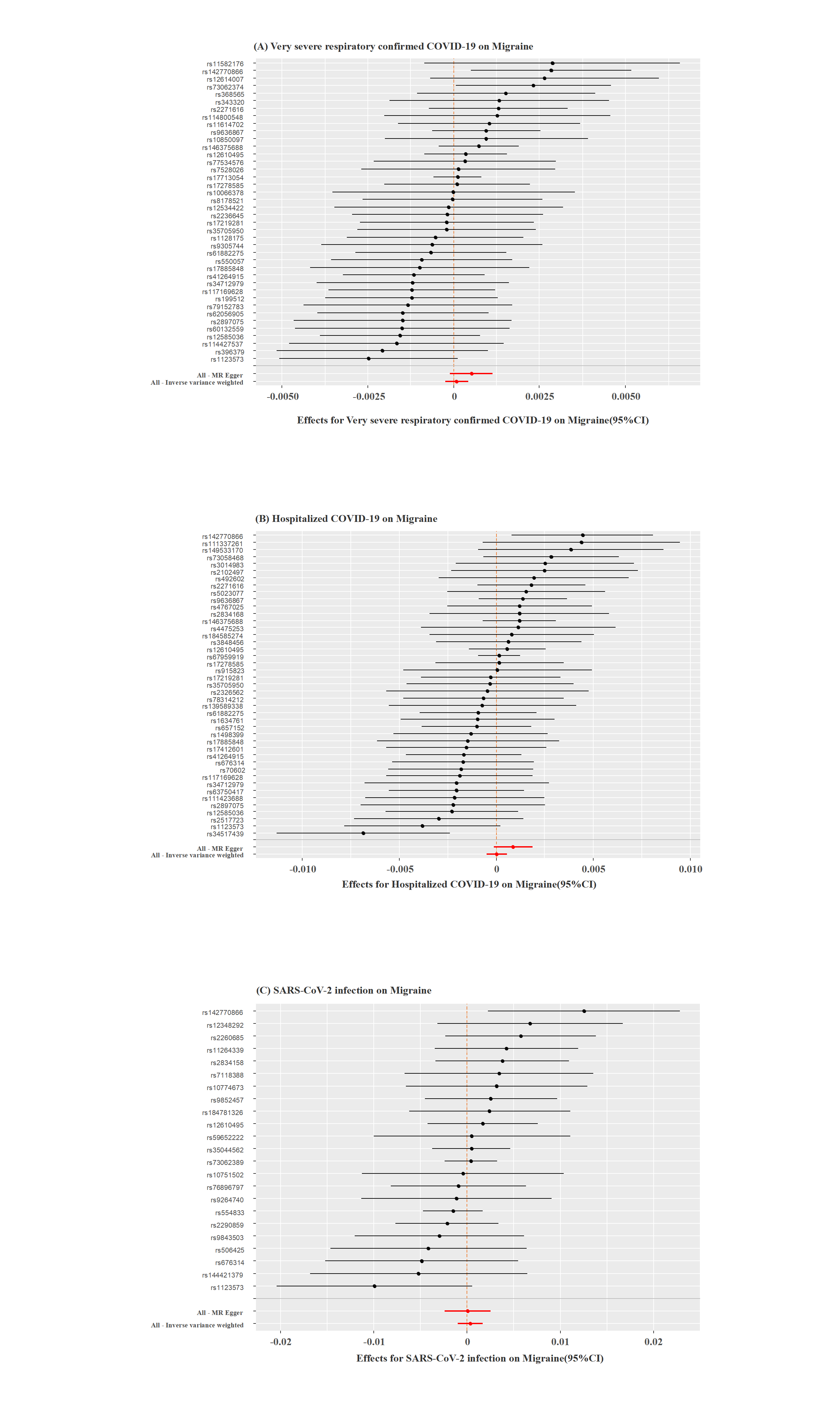

Supplement: S1 Fig — (TIF) [file pone.0308151.s001.tif]

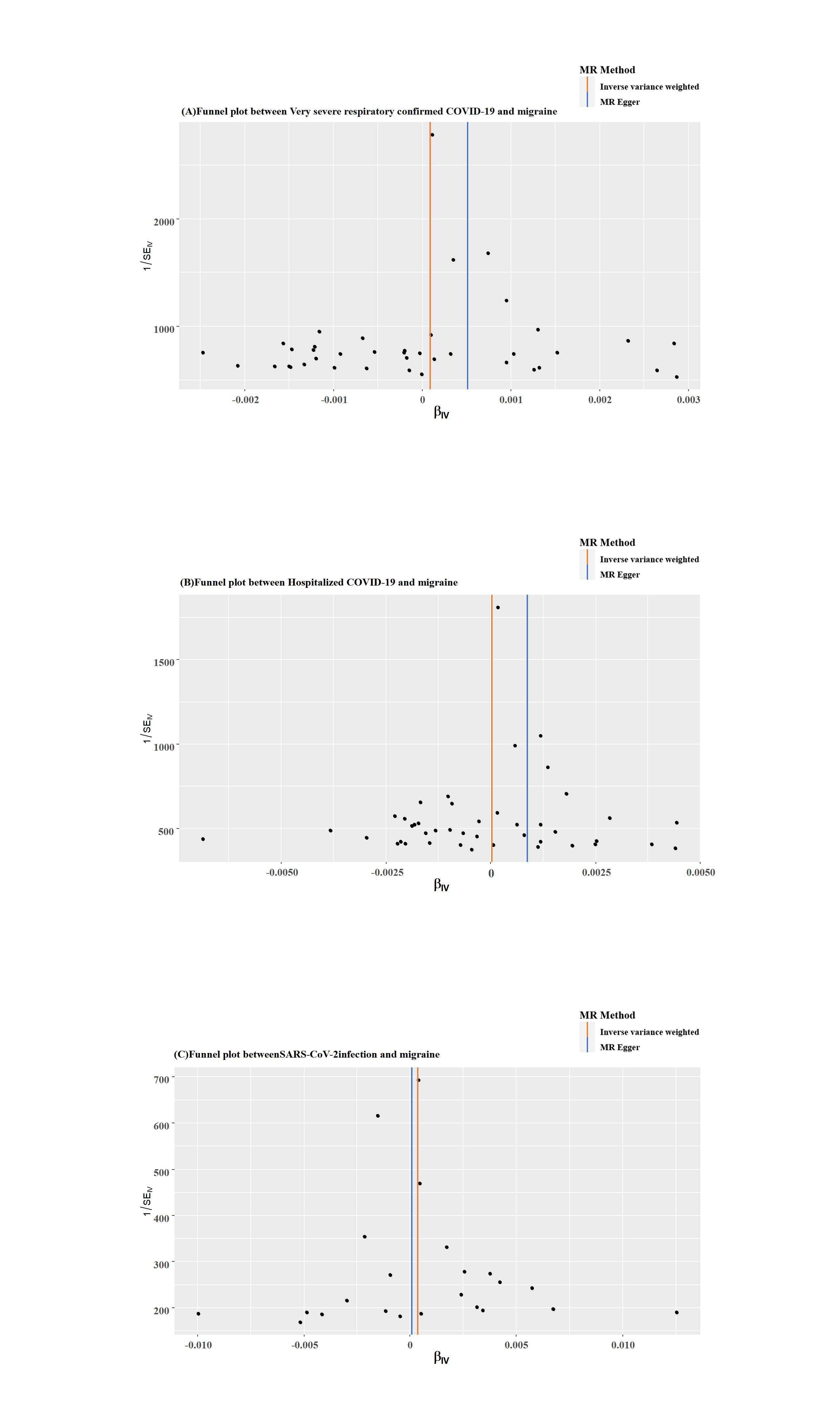

Supplement: S2 Fig — (TIF) [file pone.0308151.s002.tif]
