# Supplementary material for: COVID-19, vaccination and migraine: Causal association or epiphenomenon?
Source: PLoS One. 2024 Aug 19;19(8):e0308151. doi: 10.1371/journal.pone.0308151 (PMC11333006; doi:10.1371/journal.pone.0308151)
Supplement: S2 Table — (DOCX) [file pone.0308151.s004.docx]

**Supplementary Table 2. Details of specific single nucleotide polymorphisms for each covid-19 trait**

| **Phenotype** | **SNP** | **effect_allele** | **other_allele** | **β** | **Se** | **P-value** | **eaf** | **R^2^** | **F** |
| --- | --- | --- | --- | --- | --- | --- | --- | --- | --- |
| Very severe respiratory confirmed COVID-19 | rs10066378 | T | C | 0.11796 | 0.021006 | 1.96E-08 | 0.1159 | 0.002851572 | 77.65373568 |
| Very severe respiratory confirmed COVID-19 | rs10850097 | C | T | 0.095247 | 0.014916 | 1.71E-10 | 0.671 | 0.004005447 | 109.2023222 |
| Very severe respiratory confirmed COVID-19 | rs1123573 | A | G | -0.10621 | 0.0152 | 2.80E-12 | 0.3702 | 0.005260171 | 143.5913204 |
| Very severe respiratory confirmed COVID-19 | rs1128175 | A | G | -0.1255 | 0.017115 | 2.25E-13 | 0.7531 | 0.005857215 | 159.985356 |
| Very severe respiratory confirmed COVID-19 | rs114427537 | C | T | 0.3266 | 0.053281 | 8.80E-10 | 0.01964 | 0.004107612 | 111.9991747 |
| Very severe respiratory confirmed COVID-19 | rs114800548 | T | C | 0.23571 | 0.041161 | 1.03E-08 | 0.02819 | 0.003044125 | 82.91332392 |
| Very severe respiratory confirmed COVID-19 | rs11582176 | C | T | -0.20954 | 0.037172 | 1.73E-08 | 0.02913 | 0.002483507 | 67.60567731 |
| Very severe respiratory confirmed COVID-19 | rs11614702 | G | A | 0.10135 | 0.01383 | 2.33E-13 | 0.5069 | 0.005134933 | 140.1549452 |
| Very severe respiratory confirmed COVID-19 | rs117169628 | G | A | 0.15749 | 0.020078 | 4.37E-15 | 0.137 | 0.005864991 | 160.1989881 |
| Very severe respiratory confirmed COVID-19 | rs12534422 | C | T | 0.085624 | 0.015073 | 1.34E-08 | 0.3028 | 0.003095525 | 84.31765821 |
| Very severe respiratory confirmed COVID-19 | rs12585036 | C | T | 0.14143 | 0.017239 | 2.32E-16 | 0.2133 | 0.006712945 | 183.5169237 |
| Very severe respiratory confirmed COVID-19 | rs12610495 | A | G | 0.24167 | 0.016049 | 3.05E-51 | 0.313 | 0.025117508 | 699.619806 |
| Very severe respiratory confirmed COVID-19 | rs12614007 | G | A | 0.094133 | 0.016881 | 2.46E-08 | 0.7494 | 0.003328193 | 90.67638326 |
| Very severe respiratory confirmed COVID-19 | rs142770866 | G | A | 0.20693 | 0.025672 | 7.60E-16 | 0.08259 | 0.006488852 | 177.350703 |
| Very severe respiratory confirmed COVID-19 | rs146375688 | C | T | 0.55453 | 0.033277 | 2.39E-62 | 0.05599 | 0.032506271 | 912.3401847 |
| Very severe respiratory confirmed COVID-19 | rs17219281 | G | A | -0.17679 | 0.027005 | 5.89E-11 | 0.08179 | 0.004694481 | 128.0763711 |
| Very severe respiratory confirmed COVID-19 | rs17278585 | C | T | 0.47441 | 0.055904 | 2.14E-17 | 0.02718 | 0.011901991 | 327.082566 |
| Very severe respiratory confirmed COVID-19 | rs17713054 | G | A | 0.75584 | 0.026008 | 1.09E-185 | 0.07462 | 0.078897836 | 2325.921762 |
| Very severe respiratory confirmed COVID-19 | rs17885848 | C | T | 0.090196 | 0.015623 | 7.77E-09 | 0.3385 | 0.003643284 | 99.29240563 |
| Very severe respiratory confirmed COVID-19 | rs199512 | T | C | 0.12967 | 0.017254 | 5.68E-14 | 0.8203 | 0.004957124 | 135.2775794 |
| Very severe respiratory confirmed COVID-19 | rs2236645 | C | T | 0.17941 | 0.024951 | 6.46E-13 | 0.08481 | 0.004996681 | 136.3624828 |
| Very severe respiratory confirmed COVID-19 | rs2271616 | G | T | 0.19824 | 0.021009 | 3.87E-21 | 0.1099 | 0.007688632 | 210.3966945 |
| Very severe respiratory confirmed COVID-19 | rs2897075 | C | T | 0.087987 | 0.014375 | 9.31E-10 | 0.3757 | 0.00363163 | 98.97361334 |
| Very severe respiratory confirmed COVID-19 | rs343320 | G | A | 0.15423 | 0.027508 | 2.06E-08 | 0.07003 | 0.003098281 | 84.39296496 |
| Very severe respiratory confirmed COVID-19 | rs34712979 | G | A | -0.10951 | 0.01701 | 1.21E-10 | 0.254 | 0.004544751 | 123.9727311 |
| Very severe respiratory confirmed COVID-19 | rs35705950 | G | T | -0.1636 | 0.022785 | 6.96E-13 | 0.1085 | 0.005177829 | 141.3318476 |
| Very severe respiratory confirmed COVID-19 | rs368565 | C | T | 0.1056 | 0.014909 | 1.41E-12 | 0.4643 | 0.005547255 | 151.471813 |
| Very severe respiratory confirmed COVID-19 | rs396379 | G | T | -0.088152 | 0.01431 | 7.27E-10 | 0.5974 | 0.003737949 | 101.8820204 |
| Very severe respiratory confirmed COVID-19 | rs41264915 | A | G | -0.20626 | 0.022896 | 2.09E-19 | 0.09438 | 0.007272538 | 198.9270199 |
| Very severe respiratory confirmed COVID-19 | rs550057 | T | C | -0.11703 | 0.015941 | 2.11E-13 | 0.7261 | 0.005447696 | 148.7383841 |
| Very severe respiratory confirmed COVID-19 | rs60132559 | C | T | 0.091456 | 0.015009 | 1.11E-09 | 0.3205 | 0.003643107 | 99.28754638 |
| Very severe respiratory confirmed COVID-19 | rs61882275 | G | A | -0.12605 | 0.014659 | 8.05E-18 | 0.3432 | 0.007163019 | 195.9097209 |
| Very severe respiratory confirmed COVID-19 | rs62056905 | A | G | -0.1285 | 0.016821 | 2.18E-14 | 0.1838 | 0.004954255 | 135.1988861 |
| Very severe respiratory confirmed COVID-19 | rs73062374 | G | A | -0.16796 | 0.021324 | 3.36E-15 | 0.1332 | 0.006514257 | 178.0496104 |
| Very severe respiratory confirmed COVID-19 | rs7528026 | G | A | 0.27784 | 0.041674 | 2.61E-11 | 0.02687 | 0.004036994 | 110.0658695 |
| Very severe respiratory confirmed COVID-19 | rs77534576 | C | T | 0.31091 | 0.042425 | 2.33E-13 | 0.03393 | 0.006337119 | 173.1771459 |
| Very severe respiratory confirmed COVID-19 | rs79152783 | A | G | 0.38007 | 0.060556 | 3.47E-10 | 0.01771 | 0.005025919 | 137.1644309 |
| Very severe respiratory confirmed COVID-19 | rs8178521 | C | T | 0.11694 | 0.016929 | 4.93E-12 | 0.2498 | 0.005125375 | 139.8927239 |
| Very severe respiratory confirmed COVID-19 | rs9305744 | G | A | -0.099304 | 0.017232 | 8.27E-09 | 0.2679 | 0.003868179 | 105.4453905 |
| Very severe respiratory confirmed COVID-19 | rs9636867 | A | G | 0.18394 | 0.015088 | 3.47E-34 | 0.3312 | 0.014988872 | 413.2050568 |
| Hospitalized COVID-19 | rs111337261 | A | C | -0.14026 | 0.025413 | 3.41E-08 | 0.03854 | 0.001457943 | 71.14556728 |
| Hospitalized COVID-19 | rs111423688 | G | A | -0.074706 | 0.012144 | 7.67E-10 | 0.1491 | 0.00141611 | 69.10129503 |
| Hospitalized COVID-19 | rs1123573 | A | G | -0.068848 | 0.010432 | 4.12E-11 | 0.3773 | 0.002227298 | 108.7728025 |
| Hospitalized COVID-19 | rs117169628 | G | A | 0.10114 | 0.013651 | 1.27E-13 | 0.144 | 0.002521809 | 123.1919569 |
| Hospitalized COVID-19 | rs12585036 | C | T | 0.096736 | 0.011638 | 9.40E-17 | 0.2163 | 0.00317258 | 155.083734 |
| Hospitalized COVID-19 | rs12610495 | A | G | 0.14828 | 0.011043 | 4.17E-41 | 0.3045 | 0.009312785 | 458.0539529 |
| Hospitalized COVID-19 | rs139589338 | A | G | 0.20902 | 0.03574 | 4.96E-09 | 0.0191 | 0.001637057 | 79.90039818 |
| Hospitalized COVID-19 | rs142770866 | G | A | 0.13232 | 0.01761 | 5.74E-14 | 0.07872 | 0.002539556 | 124.0611031 |
| Hospitalized COVID-19 | rs146375688 | C | T | 0.34619 | 0.023106 | 9.53E-51 | 0.05561 | 0.012588191 | 621.2102355 |
| Hospitalized COVID-19 | rs149533170 | G | A | 0.28389 | 0.051106 | 2.78E-08 | 0.007769 | 0.001242533 | 60.62080108 |
| Hospitalized COVID-19 | rs1498399 | A | G | 0.068773 | 0.010063 | 8.24E-12 | 0.3863 | 0.002242574 | 109.5204978 |
| Hospitalized COVID-19 | rs1634761 | C | T | -0.067678 | 0.0094951 | 1.02E-12 | 0.4938 | 0.002289804 | 111.8323512 |
| Hospitalized COVID-19 | rs17219281 | G | A | -0.12453 | 0.018353 | 1.16E-11 | 0.07969 | 0.002274657 | 111.0909161 |
| Hospitalized COVID-19 | rs17278585 | C | T | 0.30519 | 0.037728 | 6.00E-16 | 0.02537 | 0.004606073 | 225.4807454 |
| Hospitalized COVID-19 | rs17412601 | T | C | -0.068259 | 0.010317 | 3.69E-11 | 0.3513 | 0.002123596 | 103.6976095 |
| Hospitalized COVID-19 | rs17885848 | C | T | 0.061114 | 0.010877 | 1.92E-08 | 0.3374 | 0.001669967 | 81.50934457 |
| Hospitalized COVID-19 | rs184585274 | G | A | 0.18763 | 0.030492 | 7.58E-10 | 0.02513 | 0.001724939 | 84.19710083 |
| Hospitalized COVID-19 | rs2102497 | T | C | 0.064371 | 0.011743 | 4.21E-08 | 0.7339 | 0.001618424 | 78.98949252 |
| Hospitalized COVID-19 | rs2271616 | G | T | 0.14396 | 0.014313 | 8.47E-24 | 0.1153 | 0.004228039 | 206.8963004 |
| Hospitalized COVID-19 | rs2326562 | C | T | 0.053141 | 0.0097363 | 4.81E-08 | 0.3814 | 0.001332539 | 65.01787298 |
| Hospitalized COVID-19 | rs2517723 | C | T | -0.062011 | 0.010388 | 2.38E-09 | 0.6093 | 0.001830805 | 89.37406348 |
| Hospitalized COVID-19 | rs2834168 | A | G | 0.063026 | 0.010271 | 8.45E-10 | 0.297 | 0.001658751 | 80.96099871 |
| Hospitalized COVID-19 | rs2897075 | C | T | 0.058531 | 0.009857 | 2.88E-09 | 0.374 | 0.001604161 | 78.29223097 |
| Hospitalized COVID-19 | rs3014983 | C | T | -0.069511 | 0.012003 | 6.99E-09 | 0.736 | 0.001877668 | 91.66607777 |
| Hospitalized COVID-19 | rs34517439 | C | A | 0.092186 | 0.015597 | 3.41E-09 | 0.1191 | 0.001783193 | 87.04564414 |
| Hospitalized COVID-19 | rs34712979 | G | A | -0.064307 | 0.011488 | 2.17E-08 | 0.2499 | 0.001550358 | 75.66226933 |
| Hospitalized COVID-19 | rs35705950 | G | T | -0.098933 | 0.015532 | 1.89E-10 | 0.1073 | 0.001875071 | 91.53903269 |
| Hospitalized COVID-19 | rs3848456 | C | A | 0.21864 | 0.028867 | 3.62E-14 | 0.03159 | 0.002924813 | 142.9367252 |
| Hospitalized COVID-19 | rs41264915 | A | G | -0.14296 | 0.015375 | 1.43E-20 | 0.09699 | 0.003579963 | 175.0692026 |
| Hospitalized COVID-19 | rs4475253 | A | G | 0.058194 | 0.010052 | 7.07E-09 | 0.3241 | 0.001483706 | 72.40463165 |
| Hospitalized COVID-19 | rs4767025 | C | T | 0.07545 | 0.010079 | 7.11E-14 | 0.6762 | 0.002492875 | 121.7749893 |
| Hospitalized COVID-19 | rs492602 | A | G | -0.055036 | 0.0095341 | 7.81E-09 | 0.4729 | 0.001510032 | 73.69125552 |
| Hospitalized COVID-19 | rs5023077 | T | C | -0.06608 | 0.0095148 | 3.79E-12 | 0.491 | 0.002182576 | 106.5839638 |
| Hospitalized COVID-19 | rs61882275 | G | A | -0.091959 | 0.0099445 | 2.30E-20 | 0.3424 | 0.00380815 | 186.2707502 |
| Hospitalized COVID-19 | rs63750417 | C | T | -0.091763 | 0.011487 | 1.37E-15 | 0.1835 | 0.002523234 | 123.261737 |
| Hospitalized COVID-19 | rs657152 | A | C | -0.10009 | 0.0097644 | 1.18E-24 | 0.6136 | 0.00475044 | 232.5816572 |
| Hospitalized COVID-19 | rs676314 | A | G | 0.077962 | 0.010055 | 8.94E-15 | 0.3246 | 0.002665051 | 130.2081249 |
| Hospitalized COVID-19 | rs67959919 | G | A | 0.49158 | 0.017513 | 2.31E-173 | 0.07742 | 0.034520383 | 1742.232502 |
| Hospitalized COVID-19 | rs70602 | T | C | 0.087112 | 0.011708 | 1.00E-13 | 0.8209 | 0.002231371 | 108.9721636 |
| Hospitalized COVID-19 | rs73058468 | C | T | -0.10968 | 0.01417 | 9.92E-15 | 0.1388 | 0.00287593 | 140.5409156 |
| Hospitalized COVID-19 | rs78314212 | C | T | 0.122 | 0.017175 | 1.22E-12 | 0.08485 | 0.002311499 | 112.8944087 |
| Hospitalized COVID-19 | rs915823 | A | C | -0.068488 | 0.012505 | 4.33E-08 | 0.2375 | 0.001698879 | 82.92289942 |
| Hospitalized COVID-19 | rs9636867 | A | G | 0.12824 | 0.010176 | 2.05E-36 | 0.3357 | 0.007334873 | 360.0505304 |
| SARS-CoV-2 infection | rs10751502 | A | G | -0.027096 | 0.0049027 | 3.26E-08 | 0.3203 | 0.000319679 | 36.11903936 |
| SARS-CoV-2 infection | rs10774673 | C | T | 0.028916 | 0.0048301 | 2.14E-09 | 0.6754 | 0.00036662 | 41.42462918 |
| SARS-CoV-2 infection | rs1123573 | A | G | -0.026386 | 0.0047693 | 3.16E-08 | 0.3789 | 0.00032769 | 37.02446159 |
| SARS-CoV-2 infection | rs11264339 | C | T | -0.035023 | 0.0045618 | 1.62E-14 | 0.4947 | 0.000613236 | 69.30702784 |
| SARS-CoV-2 infection | rs12348292 | G | A | 0.045715 | 0.0077434 | 3.55E-09 | 0.09747 | 0.000367689 | 41.5454606 |
| SARS-CoV-2 infection | rs12610495 | A | G | 0.049515 | 0.0050773 | 1.80E-22 | 0.3032 | 0.001035955 | 117.1316496 |
| SARS-CoV-2 infection | rs142770866 | G | A | 0.046809 | 0.0084387 | 2.91E-08 | 0.07867 | 0.000317624 | 35.88678073 |
| SARS-CoV-2 infection | rs144421379 | C | T | 0.064273 | 0.011743 | 4.42E-08 | 0.04086 | 0.000323793 | 36.58401159 |
| SARS-CoV-2 infection | rs184781326 | A | G | -0.06902 | 0.011679 | 3.43E-09 | 0.04715 | 0.000428042 | 48.36771232 |
| SARS-CoV-2 infection | rs2260685 | T | C | 0.033425 | 0.0046886 | 1.01E-12 | 0.4744 | 0.000557151 | 62.96485035 |
| SARS-CoV-2 infection | rs2290859 | C | T | -0.050838 | 0.0048604 | 1.32E-25 | 0.351 | 0.001177494 | 133.1538136 |
| SARS-CoV-2 infection | rs2834158 | T | C | -0.040547 | 0.0048711 | 8.50E-17 | 0.6611 | 0.000736692 | 83.2701293 |
| SARS-CoV-2 infection | rs35044562 | A | G | 0.12798 | 0.0084762 | 1.65E-51 | 0.07761 | 0.00234502 | 265.4907549 |
| SARS-CoV-2 infection | rs506425 | T | C | -0.027982 | 0.0050789 | 3.60E-08 | 0.288 | 0.000321115 | 36.28133842 |
| SARS-CoV-2 infection | rs554833 | T | C | -0.090799 | 0.0047649 | 5.88E-81 | 0.6516 | 0.003743272 | 424.3882435 |
| SARS-CoV-2 infection | rs59652222 | C | T | -0.041931 | 0.0075718 | 3.06E-08 | 0.1122 | 0.000350274 | 39.57703698 |
| SARS-CoV-2 infection | rs676314 | A | G | 0.027577 | 0.0048146 | 1.02E-08 | 0.3256 | 0.000333984 | 37.73583459 |
| SARS-CoV-2 infection | rs7118388 | A | G | 0.026551 | 0.0045331 | 4.71E-09 | 0.5057 | 0.000352432 | 39.82095276 |
| SARS-CoV-2 infection | rs73062389 | G | A | 0.20044 | 0.0098613 | 7.59E-92 | 0.05432 | 0.004127649 | 468.147071 |
| SARS-CoV-2 infection | rs76896797 | C | T | -0.061411 | 0.0078364 | 4.63E-15 | 0.1049 | 0.000708222 | 80.04981368 |
| SARS-CoV-2 infection | rs9264740 | C | T | -0.030474 | 0.0052595 | 6.87E-09 | 0.7321 | 0.000364277 | 41.15979563 |
| SARS-CoV-2 infection | rs9843503 | T | G | -0.060407 | 0.010061 | 1.92E-09 | 0.05675 | 0.000390658 | 44.1417597 |
| SARS-CoV-2 infection | rs9852457 | G | A | 0.077654 | 0.0095015 | 3.01E-16 | 0.06421 | 0.000724667 | 81.90992794 |
